# BAMITA: Bayesian Multiple Imputation for Tensor Arrays

**Published:** 2024-10-30

**Authors:** Ziren Jiang, Gen Li, Eric F. Lock

**Affiliations:** 1 Division of Biostatistics and Health Data Science, University of Minnesota; 2 Department of Biostatistics, University of Michigan

## Abstract

Data increasingly take the form of a multi-way array, or tensor, in several biomedical domains. Such tensors are often incompletely observed. For example, we are motivated by longitudinal microbiome studies in which several timepoints are missing for several subjects. There is a growing literature on missing data imputation for tensors. However, existing methods give a point estimate for missing values without capturing uncertainty. We propose a multiple imputation approach for tensors in a flexible Bayesian framework, that yields realistic simulated values for missing entries and can propagate uncertainty through subsequent analyses. Our model uses efficient and widely applicable conjugate priors for a CANDECOMP/PARAFAC (CP) factorization, with a separable residual covariance structure. This approach is shown to perform well with respect to both imputation accuracy and uncertainty calibration, for scenarios in which either single entries or entire fibers of the tensor are missing. For two microbiome applications, it is shown to accurately capture uncertainty in the full microbiome profile at missing timepoints and used to infer trends in species diversity for the population. Documented R code to perform our multiple imputation approach is available at https://github.com/lockEF/MultiwayImputation.

## 1 Introduction

Multiway data, also known as a multidimensional array or a tensor, has become more prevalent in biomedical signal processing and other diverse fields. Tensors can effectively represent real-world datasets that have multiple dimensions or aspects. However, a common challenge associated with multi-way data is missing data, which can occur either on an entry-wise basis (i.e., certain entries have missing data) or a fiber-wise basis (i.e., entire modes have missing data). For example, in this manuscript we consider microbiome studies in which the abundance of microbial taxa are collected longitudinally over several timepoints, for several subjects. The resulting data can be represented as a 3-way tensor: subjects × taxa × time points; however, the microbiome profiles (abundance across taxa) are entirely missing at several time points for several of the subjects in both studies, resulting in fiber-wise missingness. Our goal is to enable analysis of the full tensor by imputing missing data, while also accurately capturing uncertainty in the imputed fibers. Accounting for this uncertainty is particularly critical for the validity of subsequent inferences, such as for population trends in species diversity over time.

The Tucker decomposition Tucker (1966) and CANDECOMP/PARAFAC (CP) decomposition (Carroll and Chang, 1970) are two classic formulations to decompose tensor data, and can be viewed as extensions of the singular value decomposition for a matrix. The Tucker decomposition decomposes a given tensor into a core tensor multiplied by matrices corresponding to each mode. The CP decomposition, which can be viewed as a constrained case of the Tucker decomposition, decomposing the tensor into a sum of rank-1 tensors. Kolda and Bader (2009) gives a very comprehensive review of tensor decompositions and their application in various contexts.

Tensor decomposition methods are useful to uncover the underlying structure in a tensor, and have been used to impute missing elements. Acar et al. (2011) describe imputation approaches based on the CP decomposition, and Chen et al. (2013) describe imputation approaches based on a Tucker decomposition. Several related extensions incorporate penalization or smoothing into estimation of the tensor decomposition for missing data imputation (Yokota et al., 2016; Tan et al., 2013; Wu et al., 2018). An alternative approach, first proposed by Liu et al. (2012), builds upon ideas in low-rank matrix completion (Mazumder et al., 2010) by minimizing the tensor trace norm to transform the problem into a convex optimization problem.

The aforementioned imputation approaches are all deterministic, in that they only produce a single-point estimate with no uncertainty for the imputed values. A Bayesian framework is attractive in this context, because it can accommodate the collective uncertainty of the underlying factors that are combined in a low-rank decomposition. There is an extensive literature on low-rank Bayesian modeling for tensors, particularly in the regression context (Hoff, 2015; Guhaniyogi et al., 2017; Wang and Xu, 2024). However there is little work on Bayesian approaches to multiple imputation for tensors. Chen et al. (2019) proposed a Bayesian tensor decomposition approach that generalizes the Bayesian matrix factorization model of Salakhutdinov and Mnih (2008), incorporating independent and normally distributed errors. However, their implementation only provides a point estimate with no uncertainty and does not account for correlation in the residual error structure.

To our knowledge no existing tensor imputation methods perform multiple imputation, in which multiple values are simulated for the missing entries to reflect their uncertainty. However, this is often critical for applications, to accurately propagate uncertainty through subsequent analyses after imputation. As shown in our examples, imputing missing values with a point estimate and subsequently treating the values as fixed can drastically underestimate uncertainty and lead to inaccurate inference. Thus, we introduce Bayesian Multiple Imputation for Tensor Arrays (BAMITA), a flexible Bayesian model based on the CP factorization with an efficient MCMC sampling algorithm for multi-way data with missing values. Our approach enables valid inference via simulating from the posterior predictive distribution for missing values, accounting for the uncertainty of the imputed elements. Further, our approach incorporates a separable covariance structure on the error terms to account for any correlation structure that may exist on specific tensor modes. We illustrate the advantages of this approach with respect to both imputation accuracy and uncertainty calibration via simulations, and on data from two longitudinal microbiome studies.

## 2 Preliminaries

### 2.1 Notation and Background

Denote $𝓧\in R^{I_{1}\times I_{2}\times \ldots \times I_{N}}$ as an N-dimensional tensor with the length of each dimension being $I_{1},I_{2},\ldots ,I_{N}$ respectively. Let a○b denote the outer product of two vectors a and b. Denote the Kronecker product of matrices A and B as A⊗B and the *Khatri-Rao* product (which is the columnwise Kronecker product) as A⊙B. Let $X_{\left(n\right)}\in R^{I_{n}\times I_{1}I_{2}\ldots I_{n-1}I_{n+1}\ldots I_{N}}$ denote the mode-n matricization of a tensor $𝓧\in R^{I_{1}\times I_{2}\times \ldots \times I_{N}}$, yielding a matrix with $I_{n}$ rows and $\prod_{i\neq n}I_{i}$ columns. Denote the Moore-Penrose pseudoinverse of matrix A as $A^{†}$.

For our context, elements of the full tensor 𝓧 may be missing. Let $ℳ=\left\{\left(i_{1},\ldots ,i_{N}\right)\right.$: ${𝒳}_{i_{1},\ldots ,i_{N}}$ is missing} index missing entries, ${𝓧}_{\text{missing}}$ give the latent missing values in the tensor $\left\{{𝓧}_{i_{1},\ldots ,i_{N}}:\left(i_{1},\ldots ,i_{N}\right)\in ℳ\right\}$, and ${𝓧}_{\text{observed}}$ give observed values in the tensor. Our goal is to infer ${𝓧}_{\text{missing}}$ given ${𝓧}_{\text{observed}}$.

### 2.2 Tensor CANDECOMP/PARAFAC (CP) Decomposition

For a tensor $𝓧\in R^{I_{1}\times I_{2}\times \ldots \times I_{N}}$, the rank R CP decomposition (Carroll and Chang, 1970) factorizes it into a sum of component rank-one tensors that are the outer product of vectors in each dimension,

(1) $$𝓧\approx \sum_{r=1}^{R}u_{r}^{1}\circ u_{r}^{2}\circ \ldots \circ u_{r}^{N},$$

where $u_{r}^{1},u_{r}^{2},\ldots ,u_{r}^{N}$ are vectors with length $I_{1},\ldots ,I_{N}$ respectively. For i=1,...,N, define the matrix

(2) $$U^{i}=\left[\begin{array}{llll} u_{1}^{i} & u_{2}^{i} & \ldots & u_{R}^{i} \end{array}\right].$$

Then, we can express the CP decomposition in the following concise way:

$$𝓧\approx ⟦U^{1},U^{2},\ldots ,U^{N}⟧\equiv \sum_{r=1}^{R}\;u_{r}^{1}\circ u_{r}^{2}\circ \ldots \circ u_{r}^{N}.$$

By definition, the norm of each component is not identifiable, i.e.,

$$⟦X^{1}\Lambda ,X^{2},\ldots ,X^{N}⟧=⟦X^{1},X^{2}\Lambda ,\ldots ,X^{N}⟧=⟦X^{1},X^{2},\ldots ,X^{N}\Lambda ⟧$$

where $\Lambda =diag\left(\lambda_{1},\ldots ,\lambda_{R}\right)$. Thus, define the following normalized format where the columns of $U^{1},U^{2},\ldots$, and $U^{N}$ have norm 1 and λ gives the scale factors:

(3) $$𝓧\approx ⟦\lambda ;U^{1},U^{2},\ldots ,U^{N}⟧\equiv \sum_{r=1}^{R}\;\lambda_{r}u_{r}^{1}\circ u_{r}^{2}\circ \ldots \circ u_{r}^{N}.$$

We can also write express the factorization in the matricized form

(4) $$X_{\left(i\right)}=U^{i}\Lambda {\left(U^{N}⊙\ldots ⊙U^{i+1}⊙U^{i-1}⊙\ldots ⊙U^{1}\right)}^{T}$$

which will be used in our following derivations.

### 2.3 Alternating Least Squares (ALS) and EM imputation

The alternating least square (ALS) method aims to compute a CP decomposition with R components $\hat{𝓧}=\sum_{r=1}^{R}\;u_{r}^{1}\circ u_{r}^{2}\circ \ldots \circ u_{r}^{N}$ that best approximates the target tensor $𝓧\in R^{I_{1}\times I_{2}\times \ldots \times I_{N}}$ via minimizing the sum of squared residuals:

$$\underset{\hat{𝓧}}{min}\;\|𝓧-\hat{𝓧}{\|}^{2}$$

The ALS algorithm iteratively achieves this by solving the least square problem of one component (${\hat{U}}^{1}$, for example) with the other components fixed, which is

$$\begin{array}{l} {\hat{U}}^{1}=\underset{U^{1}}{argmin}{\left\|𝓧-⟦\lambda ;U^{1},U^{2},\ldots ,U^{N}⟧\right\|}_{F}\\ \;=\underset{U^{1}}{argmin}{\left\|X_{\left(1\right)}-U^{1}\Lambda {\left(U^{N}⊙\ldots ⊙U^{2}\right)}^{T}\right\|}_{F}. \end{array}$$

Solving the least square equation, we have

$$\begin{array}{l} {\hat{U}}^{1}=X_{\left(1\right)}{\left[{\left(U^{N}⊙\ldots ⊙U^{2}\right)}^{T}\right]}^{†}\\ \;=X_{\left(1\right)}\left(U^{N}⊙\ldots ⊙U^{2}\right){\left[{\left(U^{N}\right)}^{T}U^{N}*\ldots *{\left(U^{2}\right)}^{T}U^{2}\right]}^{†}. \end{array}$$

Where * is the Hadamard product which is the elementwise matrix product for two matrices with the same dimensions. We can normalize each column of ${\hat{U}}^{1}=\left[\begin{array}{llll} {\hat{u}}_{1}^{1} & {\hat{u}}_{2}^{1} & \ldots & {\hat{u}}_{R}^{1} \end{array}\right]$ to calculate $U^{1}$ and Λ where $\lambda_{r}=\left\|{\hat{u}}_{r}^{1}\right\|$ and $u_{r}^{1}={\hat{u}}_{r}^{1}/\lambda_{r}$. Then, we fix $U^{1},U^{3}\ldots ,U^{N}$ to compute $U^{2}$ in a similar way. In this way, $U^{1}\ldots ,U^{N}$ are iteratively updated until the algorithm converges (i.e., the calculated matrix is very close to the former matrix) or it attains the maximum number of iterations.

A common frequentist approach to impute missing data for tensors is an expectation-maximization (EM) algorithm, in which missing entries are iteratively updated via ALS or a similar procedure (Acar et al., 2011). Missing data are first initialized, e.g., as ${𝓧}_{i_{1},\ldots ,i_{N}}=0$ for $\left(i_{1},\ldots ,i_{N}\right)\in ℳ$ if the tensor is centered. Then, the solution to (2.3) is determined for the full tensor 𝓧, the missing entries are updated as their corresponding elements in $\hat{𝓧}$, and the process is repeated until convergence.

## 3 Bayesian Tensor Imputation Algorithm

In this section, we describe BAMITA, a Bayesian approach for the CP decomposition under different assumptions on the variance structure. We first derive the Bayesian MCMC sampling procedure under the assumption that the error (noise) term is independent and normally distributed. We then introduce another model where the error term is assumed to be normally distributed with a separable covariance structure.

### 3.1 Independent error

#### 3.1.1 The model

First, follow the notation in Section 2, assume the following model based in the CP decomposition with rank R:

(5) $$𝓧=⟦U^{1},U^{2},\ldots ,U^{N}⟧+𝓔,$$

where $𝓧\in R^{I_{1}\times I_{2}\times \ldots \times I_{N}}$, $U^{1},\ldots ,U^{N}$ defined in (2) with R components (i.e., rank R), and 𝓔 is a tensor of the error terms with the same dimensions. In the rest of this section, we treat R as fixed. In practice, the number of components R can be selected through cross-validation. Here, we assume the error terms are independent and identically distributed from a normal distribution with mean 0 and variance $\sigma^{2}$. As our goal is to have a non-informative prior that can be used in a variety of data situations, we consider an (improper) conjugate Jeffreys prior for all $U^{1},U^{2},\ldots ,U^{N}$ and $\sigma^{2}$, with,

$$p\left(U^{n}\right)\propto 1$$

for n=1,...,N and

$$p\left(\sigma^{2}\right)\propto \frac{1}{\sigma^{2}}.$$

Although the prior we impose is improper, the corresponding posterior will be proper as we will demonstrate that all the conditional distributions for the posterior are proper. Moreover, the model is invariant to the relative scale of the $U^{i}$; one can scale the individual factors as in (3), but this will not affect posterior inference for the low-rank structure and missing data, which is our primary focus.

#### 3.1.2 Gibbs Sampler with full data

Here we give full conditionals for the Gibbs sampling procedure for fully observed data. Note that, given $U^{2},\ldots ,U^{N}$ and $\sigma^{2}$, the model can be expressed as:

(6) $$X_{\left(1\right)}=U^{1}{\left(U^{N}⊙U^{\left(N-1\right)}⊙\ldots ⊙U^{2}\right)}^{T}+E_{\left(1\right)}$$

where $X_{\left(1\right)}\in R^{I_{1}\times I_{2}\ldots I_{N}}$ is the mode-1 matricization of the tensor $\hat{𝓧}=⟦U^{1},U^{2},\ldots ,U^{N}⟧$ and $E_{\left(1\right)}$ is the mode-1 matricization of the error tensor 𝓔 with the same dimension as $X_{\left(1\right)}$. Let $A_{\left(1\right)}=(U^{N}⊙U^{\left(N-1\right)}⊙\ldots ⊙U^{2})$ where the subscript 1 indicate that it is the Khatri-Rao product without matrix $U^{1}$. We partition $X_{\left(1\right)}$, $U^{1}$, and $E_{\left(1\right)}$ along their row, i.e.,

$$X_{\left(1\right)}={\left[\begin{array}{llll} {\left(x_{1\cdot }^{1}\right)}^{T} & {\left(x_{2\cdot }^{1}\right)}^{T} & \ldots & {\left(x_{I_{1}\cdot }^{1}\right)}^{T} \end{array}\right]}^{T}$$

and,

$$U^{1}={\left[\begin{array}{llll} {\left(u_{1\cdot }^{1}\right)}^{T} & {\left(u_{2\cdot }^{1}\right)}^{T} & \ldots & {\left(u_{I_{1}\cdot }^{1}\right)}^{T} \end{array}\right]}^{T}$$

and,

$$E_{\left(1\right)}={\left[\begin{array}{llll} {\left(\epsilon_{1\cdot }^{1}\right)}^{T} & {\left(\epsilon_{2\cdot }^{1}\right)}^{T} & \ldots & {\left(\epsilon_{I_{1}\cdot }^{1}\right)}^{T} \end{array}\right]}^{T}.$$

Since each element in the error term is assumed to be independent and normally distributed, we have the following Bayesian linear regression model

(7) $$x_{i\cdot }^{1}=u_{i\cdot }^{1}A_{\left(1\right)}+\epsilon_{i\cdot }^{1}$$

for $i=1,\ldots ,I_{1}$ where $\epsilon_{i\cdot }^{1}$ be i.i.d. normal distributed with mean 0 and variance $\sigma^{2}$. Then, with the Jeffreys priors $p\left(u_{i\cdot }^{1}\right)\propto 1$ and $p\left(\sigma^{2}\right)\propto \frac{1}{\sigma^{2}}$, the posterior distribution for $u_{i}^{1}$. is a normal distribution with mean ${\left(A_{\left(1\right)}^{T}A_{\left(1\right)}\right)}^{-1}A_{\left(1\right)}^{T}x_{i\cdot }^{1}$ and covariance matrix $\sigma^{2}{\left(A_{\left(1\right)}^{T}A_{\left(1\right)}\right)}^{-1}$. Therefore, we have the following Gibbs sampling algorithm for fully observed data:

- Given $U^{2},\ldots ,U^{N}$ and $\sigma^{2}$, draw
$${\left.U^{1}={\left[\begin{array}{llll} {\left(u_{1\cdot }^{1}\right)}^{T} & {\left(u_{2\cdot }^{1}\right)}^{T} & \ldots & \left(u_{I_{1}\cdot }^{1}\right. \end{array}\right)}^{T}\right]}^{T}$$
  with
$$u_{i\cdot }^{1}∼N({\left(A_{\left(1\right)}^{T}A_{\left(1\right)}\right)}^{-1}A_{\left(1\right)}^{T}x_{i\cdot }^{1},\sigma^{2}{\left(A_{\left(1\right)}^{T}A_{\left(1\right)}\right)}^{-1})$$
  for $i=1,\ldots ,I_{1}$ where $A_{\left(1\right)}=(U^{N}⊙U^{\left(N-1\right)}⊙\ldots ⊙U^{2})$.
- Draw $U^{2},\ldots ,U^{N}$ similarly:
$$U^{n}={\left[\begin{array}{llll} {\left(u_{1\cdot }^{n}\right)}^{T} & {\left(u_{2\cdot }^{n}\right)}^{T} & \ldots & {\left(u_{I_{n}\cdot }^{n}\right)}^{T} \end{array}\right]}^{T}$$
  with
$$u_{i\cdot }^{n}∼N({\left(A_{\left(n\right)}^{T}A_{\left(n\right)}\right)}^{-1}A_{\left(n\right)}^{T}x_{i\cdot }^{1},\sigma^{2}{\left(A_{\left(n\right)}^{T}A_{\left(n\right)}\right)}^{-1})$$
  for $i=1,\ldots ,I_{n}$ where $A_{\left(n\right)}=(U^{N}⊙\ldots ⊙U^{\left(n+1\right)}⊙U^{\left(n-1\right)}⊙\ldots ⊙U^{1})$.
- Given $U^{1},\ldots ,U^{N}$, draw $\sigma^{2}$ via its inverse-gamma full conditional IG($\alpha =\left(I_{1}\cdot \ldots \right.\left.{\cdot I}_{N}\right)/2$, $\beta =(\|𝓧-\hat{𝓧}{\|}_{F})/2$) where $\hat{𝓧}$ is calculated using the simulated $U^{1},\ldots ,U^{N}$ from the previous steps, and $\|\cdot {\|}_{F}$ be the Frobenius norm.

#### 3.1.3 Missing data imputation

We propose to impute the missing entries ${𝓧}_{i_{1},\ldots ,i_{N}}$, $\left(i_{1},\ldots ,i_{N}\right)\in ℳ$ with simulated values from their posterior predictive distribution $p(Vec({𝓧}_{\text{missing}})|Vec({𝓧}_{\text{observed}}))$ for each MCMC iteration. Note that, if we assume the error terms are independently distributed, the conditional distribution will simply be determined by the posterior distribution for the error variance and low-rank structure for the missing entries. The missing entries will be imputed according to their posterior sampling. Given a partially observed tensor 𝓧 and number of modes R, our Bayesian multiple imputation algorithm for normal i.i.d error is given in Algorithm 1.

**Algorithm 1:** Bayesian multiple imputation with normal i.i.d error

1:	Impute the missing elements as ${𝓧}_{i_{1},\ldots ,i_{N}}=0$, $\left(i_{1},\ldots ,i_{N}\right)\in ℳ$.
2:	Set the initial value of ${\left({\hat{U}}^{1}\right)}^{0},\ldots ,{\left({\hat{U}}^{N}\right)}^{0}$ as either randomly generated number or the result of the frequentist EM algorithm. Set the initial value of ${\left({\overset{ˆ}{\sigma }}^{2}\right)}^{0}∼IG(\alpha =(I_{1}\cdot \ldots \cdot I_{N})/2,\;\beta =(∥𝓧-\hat{𝓧}{∥}_{F})/2)$ where $\hat{𝓧}$ is calculated using the initial value of ${\left({\hat{U}}^{1}\right)}^{0},\ldots ,{\left({\hat{U}}^{N}\right)}^{0}$.
3:	**for** r=1,…,B-th MCMC iteration **do**
4:	Sample ${\left({\hat{U}}^{1}\right)}^{r},\ldots ,{\left({\hat{U}}^{N}\right)}^{r}$, and ${\left({\overset{ˆ}{\sigma }}^{2}\right)}^{r}$ with the previous posterior distributions.
5:	Calculate the underlying structure of 𝓧 as ${\tilde{𝓧}}^{r}=⟦{\left(U^{1}\right)}^{r},\ldots ,{\left(U^{N}\right)}^{r}⟧$
6:	Calculate ${\hat{𝓧}}^{r}$ where missing elements ${𝓧}_{i_{1}\ldots i_{N}}$, $\left(i_{1}\ldots i_{N}\right)\in ℳ$ are imputed following $N({\tilde{𝓧}}_{i_{1},\ldots ,i_{N}}^{r},{\left({\overset{ˆ}{\sigma }}^{2}\right)}^{r})$
7:	**end for**
8:	Impute the missing elements ${𝓧}_{i_{1}\ldots i_{N}},\left(i_{1},\ldots ,i_{N}\right)\in ℳ$ as mean value of $\left\{{\tilde{𝓧}}_{i_{1},\ldots ,i_{N}}^{b},\ldots ,{\tilde{𝓧}}_{i_{1},\ldots ,i_{N}}^{B}\right\}$where b be the burn-in value of the Gibbs sampler.

In practice, the rank R can be determined by cross-validation. In Algorithm 2, we describe a general cross-validation procedure for choosing R using the mean squared error (MSE) of the imputed held-out elements.

**Algorithm 2:** Selecting number of the component with cross validation

1:	Randomly divide the observed elements into K equal folds with index ${ℱ}_{1},\ldots ,{ℱ}_{K}$.
2:	**for** r=1,…,R number of components **do**
3:	**for** k=1,…,K-th fold **do**
4:	Hold out the elements in the k-th fold ${𝓧}_{i_{1},\ldots ,i_{N}}=0,\;\left(i_{1},\ldots ,i_{N}\right)\in {ℱ}_{k}$ as missing.
5:	Run the corresponding Bayesian imputation algorithm (Algorithm 1 for independent error) and get the imputed value ${\hat{𝓧}}_{i_{1},\ldots ,i_{N}}$, $\left(i_{1},\ldots ,i_{N}\right)\in {ℱ}_{k}$ for the held-out data.
6:	Calculate the mean squared error (MSE) for the imputation over the held-out data. $\delta_{k}=\sum_{\left(i_{1},\ldots ,i_{N}\right)\in {ℱ}_{k}}{\left({\hat{𝓧}}_{i_{1},\ldots ,i_{N}}-{𝓧}_{i_{1},\ldots ,i_{N}}\right)}^{2}$.
7:	**end for**
8:	Calculate the average MSE with number of component equals r as ${\overset{‾}{\delta }}^{r}=\frac{1}{K}\sum_{k=1}^{K}\delta_{k}$.
9:	**end for**
10:	Select the optimal number of components with the smallest average MSE.

## 3.2 Correlated error

### 3.2.1 The model

For many tensor applications, data have a residual correlation along some of the modes that is not efficiently captured by the low-rank decomposition. For example, in our longitudinal infant gut microbiome data, observations are assumed to be correlated across time intervals for the same participants, and abundances are correlated across the taxa. Therefore, instead of assuming the errors are independent, here we assume the errors are normally distributed but correlated. For simplicity, we assume the error tensor in (5) has the separable covariance structure described by Hoff (2011):

(8) $$𝓔∼N_{I_{1}\times I_{2}\times \ldots \times I_{N}}\left(0,\Sigma_{1},\ldots ,\Sigma_{N}\right),$$

where the tensor normal distribution $N_{I_{1}\times I_{2}\times \ldots \times I_{N}}\left(0,\Sigma_{1},\ldots ,\Sigma_{N}\right)$ is defined according to its n-th mode unfolding ${𝓔}_{\left(n\right)}$ which follows a matrix normal distribution:

$${𝓔}_{\left(n\right)}∼N_{I_{n}\times I_{-n}}\left(0,\Sigma_{n},\Sigma_{-n}\right)$$

where $I_{-n}=\prod_{i=1,\ldots ,N;i\neq n}\;I_{i}$ and $\Sigma_{-n}=\Sigma_{1}⊗\Sigma_{2}⊗\ldots \Sigma_{n-1}⊗\Sigma_{n+1}⊗\ldots \Sigma_{N}$. The vectorization of the tensor then follows a multivariate normal distribution:

$$Vec(𝓔)∼N_{I_{1}\cdot I_{2}\ldots \cdot I_{N}}\left(0,\Sigma_{N}⊗\Sigma_{N-1\cdots }⊗\Sigma_{1}\right).$$

Compared with estimating the entire unrestricted covariance matrix Cov(Vec(𝓔)) which can be unrealistic due to its potentially high dimension, using a separable covariance structure provides a more stable and parsimonious way of modeling the correlation along different modes. Moreover, it is also more interpretable for the covariance matrices $\Sigma_{i}$ for each mode, e.g., a covariance for time points and a covariance for taxa.

Similar to the independent error, we use a non-informative prior to make our algorithm broadly accommodating, with the uniform prior on each $U^{1},\ldots ,U^{N}$

$$U^{n}\propto 1$$

and inverse-Wishart prior on $\Sigma_{1},\ldots ,\Sigma_{N}$,

$$P\left(\Sigma_{n}\right)∼IW\left(Diag\left(I_{n}\right),\left(I_{n}+2\right)\right).$$

In practice, the set of inverse-Wishart priors for each dimension depends on the specific scientific context of the application. In our implementation, the first dimension is considered the sample dimension and samples are independent. Thus, we set $\Sigma_{1}$ to the identity and use a inverse-Wishart prior on the other dimensions.

### 3.2.2 Bayesian Gibbs Sampler for full data

Similar to the independent case, our model facilitates a conjugate MCMC sampling procedure for fully observed data:

- Given $U^{2},\ldots ,U^{N}$ and $\Sigma_{2},\ldots ,\Sigma_{N}$, sample $\Sigma_{1}$ with
$$\Sigma_{1}|U^{1},U^{2},\ldots ,U^{N},\Sigma_{2},\ldots ,\Sigma_{N}∼IW\left(S_{n},I_{n}+2+I_{-n}\right)$$
  where $S_{n}=Diag\left(I_{n}\right)+{\left({\left({\tilde{A}}^{T}\tilde{A}\right)}^{-1}{\tilde{A}}^{T}{\tilde{X}}_{\left(1\right)}\tilde{A}-{\tilde{X}}_{\left(1\right)}\right)}^{T}({\left({\tilde{A}}^{T}\tilde{A}\right)}^{-1}{\tilde{A}}^{T}{\tilde{X}}_{\left(1\right)}\tilde{A}-{\tilde{X}}_{\left(1\right)})$, $\tilde{A}=A\Sigma_{-1}^{-1/2}$, and ${\tilde{X}}_{\left(1\right)}=X_{\left(1\right)}\Sigma_{-1}^{-1/2}$.
- Given $\Sigma_{1}$ and $U^{2},\ldots ,U^{N}$ and $\Sigma_{2},\ldots ,\Sigma_{N}$, sample $U^{1}$ with
$$U^{1}|U^{2},\ldots ,U^{N},\Sigma_{1},\ldots ,\Sigma_{N}∼MatrixNormal({\left({\tilde{A}}^{T}\tilde{A}\right)}^{-1}{\tilde{A}}^{T}{\tilde{X}}_{\left(1\right)},{\left({\tilde{A}}^{T}\tilde{A}\right)}^{-1},\Sigma_{1})$$
- Sample $U^{2},\Sigma_{2};\ldots ;U^{N},\Sigma_{N}$ in a similar way.

### 3.2.3 Missing data imputation

The multiple imputation algorithm for ${𝓧}_{\mathrm{missing}}$ given ${𝓧}_{\mathrm{observed}}$ proceeds somewhat differently for a separable covariance structure than that under independence in Algorithm 1, because the observed values provide additional information to inform missing elements beyond the low rank signal. Let $\mu_{m}$ and $\mu_{o}$ be the vectorized mean of the missing and observed entries, respectively, given by the low-rank structure (2). Under the multivariate normal error term with separable covariance structure, the conditional posterior predictive distribution of the missing entries will be

(9) $$p(Vec({𝓧}_{\text{missing}})|Vec({𝓧}_{\text{observed}}))∼N(\mu_{\text{missing|observed}},\Sigma_{\text{missing|observed}})$$

where

$$\mu_{\text{missing}|\text{observed}}=\mu_{m}+\Sigma_{12}{\left(\Sigma_{22}\right)}^{-1}({𝓧}_{\text{observed}}-\mu_{o})$$

and

$$\Sigma_{\text{missing}|\text{observed}}=\Sigma_{11}-\Sigma_{12}{\left(\Sigma_{22}\right)}^{-1}\Sigma_{21}.$$

Here, $\Sigma_{11}$, $\Sigma_{12}$, $\Sigma_{21}$, $\Sigma_{22}$ partition the full covariance matrix of the vectorized tensor according to the missing indices (i.e., $\Sigma_{11}=Cov\left(\mu_{m},\mu_{m}\right)$ and $\Sigma_{21}=Cov\left(\mu_{o},\mu_{m}\right)$, etc).

**Algorithm 3:** Bayesian multiple imputation with correlated error

1:	Impute the missing elements in 𝓧 as 0.
2:	Set the initial value of ${\left({\hat{U}}^{1}\right)}^{0},\ldots ,{\left({\hat{U}}^{N}\right)}^{0}$ as either randomly generated number or the result of the frequentist EM algorithm. Set the initial value of ${\left({\overset{ˆ}{\Sigma }}_{n}\right)}^{0}$ as standard diagonal matrix with dimension $I_{n}$ for n=1,…,N.
3:	**for** r=1,…,B-th MCMC iteration **do**
4:	Sample ${\left({\hat{U}}^{1}\right)}^{r},{\left({\overset{ˆ}{\Sigma }}_{1}\right)}^{r};\ldots ;{\left({\hat{U}}^{N}\right)}^{r},{\left({\overset{ˆ}{\Sigma }}_{N}\right)}^{r}$ with the previous posterior distributions.
5:	Calculate the underlying structure of 𝓧 as ${\tilde{𝓧}}^{r}=⟦{\left(U^{1}\right)}^{r},\ldots ,{\left(U^{N}\right)}^{r}⟧$
6:	Partition the mean $Vec\left({\tilde{𝓧}}^{r}\right)$ into $Vec{\left({\tilde{𝓧}}^{r}\right)}_{o}$ and $Vec{\left({\tilde{𝓧}}^{r}\right)}_{m}$. Partition${\tilde{\Sigma }}^{r}={\overset{ˆ}{\Sigma }}_{N}^{r}⊗{\overset{ˆ}{\Sigma }}_{N-1}^{r}\cdots ⊗{\overset{ˆ}{\Sigma }}_{1}^{r}$ into $\Sigma_{11}^{r}$, $\Sigma_{12}^{r}$, $\Sigma_{21}^{r}$, and $\Sigma_{22}^{r}$ according to the corresponding missingness of the elements.
7:	Simulate ${\hat{𝓧}}_{\text{missing}}^{r}$ according to the posterior predictive distribution described in 9 and get ${\hat{𝓧}}^{r}$ where the missing entries are imputed with ${\hat{𝓧}}_{\text{missing}}^{r}$.
8:	end for
9:	Impute the missing entries ${𝓧}_{i_{1}\ldots i_{N}}$ as mean value of $\left\{{\tilde{𝓧}}_{i_{1},\ldots ,i_{N}}^{b},\ldots ,{\tilde{𝓧}}_{i_{1},\ldots ,i_{N}}^{B}\right\}$ where b be the burn-in value of the Gibbs sampler.

Given a observed tensor ${𝓧}_{\mathrm{observed}}$ and number of modes N, we now describe an algorithm to simulate from the posterior predictive distribution, $p({𝓧}_{\mathrm{missing}}|{𝓧}_{\mathrm{observed}})$, for missing entries. Our Bayesian multiple imputation algorithm for error term with separable covariance structure is described in Algorithm 3.

Note that, in our implementation, to be consistent with our data application, the first dimension is considered the sample dimension and samples are independent. Thus, we set $\Sigma_{1}$ to the identity and use an inverse-Wishart prior on $\Sigma_{2},\ldots ,\Sigma_{N}$. In practice, whether or not the full covariance is modeled for a given dimension can depend on the context of the application.

## 4 Simulation

To evaluate the performance of our proposed Bayesian tensor imputation methods, we conducted a series of simulation experiments. Since we propose two Bayesian imputation methods for multiway data, we aim to use the simulation experiments to evaluate:

1. The performance of the Bayesian independent imputation algorithm in terms ofrank selection under the cross-validation.
2. The performance of the Bayesian multiple imputation algorithms with independentor correlated error in terms of the imputed data entries.
3. The performance of the Bayesian multiple imputation algorithms with independent or correlated error in terms of fiber-wise imputation and inferring uncertainty for functions of the fibers.

For each simulation condition, we run 100 experiments with two independent MCMC chains. All the results are calculated as the median of the converged experiments of the Bayesian algorithm, where the convergence is evaluated using a composite version of the scale reduction factor defined as:

$$SRF=\frac{2\sum_{i=1}^{n_{1}+n_{2}}{\left(X_{i}\;-\;\overset{‾}{X}\right)}^{2}/\left(n_{1}\;+\;n_{2}\;-\;1\right)}{\sum_{i=1}^{n_{1}}{\left(X_{1i}\;-\;{\overset{‾}{X}}_{1}\right)}^{2}/\left(n_{1}\;-\;1\right)\;+\;\sum_{i=1}^{n_{2}}{\left(X_{2i}\;-\;{\overset{‾}{X}}_{2}\right)}^{2}/\left(n_{2}\;-\;1\right)}$$

where $\left\{X_{11},X_{12},\ldots ,X_{1n_{1}}\right\}$ and $\left\{X_{21},X_{22},\ldots ,X_{2n_{2}}\right\}$ be the posterior samples of the two separate chains and $\left\{X_{1},X_{2},\ldots ,X_{n_{1}+n_{2}}\right\}$ is the combination of those chains.

### 4.1 Simulation study 1: Rank selection and independent error

For our first study, simulated data 𝓧 of dimension $I_{1}\times I_{2}\times I_{3}$ is generated following (5), where $⟦U^{\left(1\right)},U^{\left(2\right)},U^{\left(3\right)}⟧$ has a rank-3 underlying structure and 𝓔 has independent normal error with mean 0 and variance 1. Each $U^{\left(i\right)}$, i=1,2,3 is a matrix of dimension $I_{i}\times 3$ whose elements are drawn from a standard normal distribution. To demonstrate the performance of our algorithm in different scales of dimensions, we considered three scenarios: (10×10×10), (20×20×20), and a higher-dimensional imbalanced scenario (10×100×1000). Different missing patterns and proportions of the tensor elements are also considered. For an entry-wise missing scenario, each element of the tensor is randomly set to be missing with the probability of 0.2, 0.5, or 0.7 (yielding missing proportions of 20%, 50%, or 70%, respectively). For a fiber-wise missing scenario, the elements for the entire fiber of the third mode are randomly set to be missing with the corresponding probability.

We run our algorithms with the assigned number of components (rank) equal to 1,2,3,4, or 5 (the true rank is 3) and select the rank according to cross-validation. The validation set is composed of the elements randomly drawn from the observed elements (i.e., not the elements which are set to be missing) of the simulated tensor data. The rank is selected based on the mean squared error (MSE) over the validation set (25% of all the observed elements). For the fiber-wise missing condition, the validation set is also randomly assigned for the entire fiber to be consistent. We evaluate the performance of our Bayesian independent imputation algorithm based on the mean squared error (MSE) and the coverage rate of the 95% credible interval with the true rank and the selected rank. The upper and lower bounds for the 95% credible interval for each element are calculated using the 0.025 and 0.975 quantiles of the MCMC samples. The MSE and coverage are calculated as median values over all the missing elements.

We compare the performance of our Bayesian multiple imputation with independent error against the frequentist EM algorithm with the true rank described in Section 2.3. Results are presented in Table 1. The MSE for the true rank and the selected rank are very close across conditions, which indicates the reasonable performance of rank selection via cross-validation. In fact, the cross-validation selects the true rank in most of the experiments except for the scenarios of 10 × 10 × 10 dimension and 70% missingness.

Coverage rates for credible intervals are all approximately 95%, so uncertainty is correctly inferred. Morevoer, our Bayesian independent imputation method performs better than the EM algorithm in terms of the MSE in most of the scenarios. When the dimension of the tensor is (10 × 100 × 1000), the two algorithms have similar performance. This matches our expectation since when the total sample size is large enough (for the element-wise missing), the results solved by the frequentist EM algorithm are generally similar to the posterior mean for the Bayesian model with flat priors and independent error. MCMC convergence for the fixed number of iterations is generally achieved, but less so for scenarios with 70% missing fibers.

### 4.2 Simulation study 2: Tensor imputation and correlated error

The second simulation study evaluates the performance of our Bayesian imputation algorithm with correlated error, in terms of the imputed entries or fibers in the tensor. Similar to the first simulation, we generate 𝓧 according to (5) with $⟦U^{\left(1\right)},U^{\left(2\right)},U^{\left(3\right)}⟧$ as rank-3 underlying structure. To better mimic the condition of our application data, we simulate 𝓧 with dimensions 10 × 10 × 10, 20 × 20 × 20, and 65 × 168 × 6. The error terms 𝓔 are generated with separable covariance structure:

$$𝓔∼N_{I_{1}\times I_{2}\times I_{3}}(0,\Sigma_{1},{\left(\Sigma_{2}\Sigma_{2}\right)}^{T},\left(\Sigma_{3}\Sigma_{3}^{T}\right))$$

where $\Sigma_{i}$, i=2,3 are the $I_{2}\times I_{2}$ and $I_{3}\times I_{3}$ covariance matrix with diagonal elements being 0.9 and other elements randomly set to be either 0.3 or −0.3 with equal probability. Following our applications, $\Sigma_{1}$ is set to be the $I_{1}\times I_{1}$ diagonal matrix (i.e., there is no correlation structure among the first dimension) with diagonal elements being 0.5. The different missing patterns and proportions of the tensor elements are the same as in the first simulation study. In this simulation study the rank is set to be 3 and the performance of each algorithm is directly evaluated over the missing elements.

The missing value is imputed using the posterior mean, and we calculate the mean squared error (MSE) and the coverage rate for the 95% credible interval of the missing elements for the two Bayesian methods. For the frequentist EM method, we calculate the MSE for the missing elements as a comparison. We also compare with the missForest method (Stekhoven and Bühlmann, 2012) applied to the matricized data along the first mode, as a general approach that does not assume low-rank tensor structure.

The results can be seen in Table 2. For the 70% fiber-wise missing with a dimension of (10 × 10 × 10), we do not include the results as only 4% of the total experiments converged. We can see that the Bayesian correlated imputation algorithm outperforms the Bayesian independent imputation algorithm and the frequentist EM algorithm in most of the scenarios in terms of the MSE except for the 70% fiber-wise missing case with dimension (65 × 168 × 6). The coverage of the correlated algorithm is also comparable to the independent algorithm, and both have coverage close to the nominal rate of 95%.

### 4.3 Simulation study 3: Imputation for function of a fiber

In the third simulation study, we examine whether an arbitrary function of the entire fiber can be reasonably captured when the data is generated with a correlation structure. This is motivated by our data applications where, instead of focusing on the imputed data entries, we are interested in the alpha diversity calculated as a function of the entire mode-2 fiber for each subject and each time point (see section 5 for more detail). We argue that although the MSE of each imputed element is mostly adopted as the metric for evaluating the imputation performance, the “structure” of the imputed data slice (i.e., fiber) is also of interest for downstream analyses of the tensor.

The data generating mechanism is similar to that of the second simulation study except that the covariance matrix $\Sigma_{1}$ and $\Sigma_{3}$ are now identity matrices with dimensions $I_{2}$ and $I_{3}$ respectively, and $\Sigma_{2}$ is a matrix with diagonal elements being 1 and other non-diagonal elements being 0.15. Under the data-generating mechanism, we do not expect the Bayesian correlated algorithm to outperform the independent algorithm in terms of the point-wise imputation error since the other modes (modes 1 and 3) are uncorrelated. However, with the additional model of the covariance structure for mode 2, our correlated algorithm may better capture the variation of the function of the imputed mode 2 fiber. To evaluate this, for each imputed fiber $\hat{𝓧}[i,\cdot ,k]$, we calculate the linear predictor $\beta_{b}\hat{𝓧}[i,\cdot ,k]$ with randomly generated coefficient $\beta_{b}$, b=1,…,100. Then we evaluate the mean MSE and coverage (only for fiber-wise missing) for the linear predictors over the 100 randomly generated coefficients $\beta_{b}$. The results are displayed as“MSE(Fiber)” in Table 3.

The results are shown in Table 3, from which we can see that, although the Bayesian independent algorithm has a relatively good coverage rate for each imputed element, the mean coverage for the random linear combination of each imputed fiber is not ideal, especially in the high-dimension cases. The Bayesian correlated algorithm, on the other hand, has substantially better coverage performance in terms of the imputed fiber.

## 5 Longitudinal microbiome applications

### 5.1 Infant gut microbiome application

The gut houses a rich array of microbial organisms, presenting a diverse landscape. This dynamic ecosystem holds potential as significant indicators for both digestive and broader health conditions. We consider a longitudinal study of the gut microbiome on 52 infants in the neonatal intensive care unit (NICU), where stool samples were collected over the first 3 months of life (Cong et al., 2017). Using 16s rRNA sequencing technology, we obtained microbiome data, which we aggregated to the genus level, yielding 152 distinct genera. Employing standard preprocessing techniques, we addressed zero values by introducing pseudo counts, transformed the data into compositional profiles, and applied the centered log-ratio (clr) transformation. Data were aggregated every 5 consecutive days, yielding 30 time intervals. Consequently, we obtained a tensor data array with dimensions of 52 × 152 × 30. However, due to the unavailability of samples from every infant at every time point, the tensor array exhibits a fiber-wise missing structure, with approximately 71% of the samples being absent. Our objective is to employ BAMITA to address missing values and assess the dynamic diversity in the microbiome over this population. Before applying our algorithm to this dataset, we first check the suitability of the normality assumption and the separable covariance assumption. Results are presented in Appendix A, and suggest that the two assumptions are reasonable for these data. The microbiome diversity is often measured using alpha diversity which refers to diversity on a local scale. (Thukral, 2017). Here, we compute diversity using the Shannon-Wiener index Shannon (1948). For a given fiber of the data $X_{i,k}$, the Shannon alpha diversity is calculated as $-\sum_{j=1}^{152}\;p_{j}\;log\left(p_{j}\right)$ where $p_{j}=\frac{exp\left({ClrX}_{i,j,k}\right)}{\sum_{j=1}^{152}exp({ClrX}_{i,j,k})}$ is the proportion of element j in the sample.

We impute the missing fibers of the microbiome tensor array with the two proposed Bayesian algorithms and the EM algorithm approach. For the Bayesian multiple imputation algorithm with separable covariance structure error, we assume independence in the first dimension (the 52 infants) but account for correlation across time and genera. The performance of the different algorithms is evaluated through cross-validation. For each of 200 simulation experiments, we randomly hold an additional 25% of the observed fibers out and calculate the mean squared error of the imputed values and the true observed value. Shannon diversity is also computed for the missing fibers at each MCMC iteration, and they are compared to the observed diversity measures for the validation set. For the Bayesian imputation with covariance structure (BAMITA Correlated), we also evaluate the MSE for the estimated low-rank structure, i.e., $⟦\left(U^{1}\right),\ldots ,\left(U^{N}\right)⟧$, which represent the imputation without adjustment of covariance structure.

The results are summarized in Table 4. The low-rank Bayesian imputation approach with correlation structure performs substantially better than other approaches with respect to MSE for both imputed values in the fiber and Shannon diversity. This suggests that the data have a strong low-rank structure, with substantial correlation in the residual covariance. Moreover, while coverage rates are appropriate for the fiber-wise entries under both models, coverage for Shannon diversity is much higher for the correlated model. This illustrates the notion that accurately inferring uncertainty in the marginal distribution for the entries of an array does not imply uncertainty will be accurately inferred for multivariate functions that are used in downstream analysis. The MSE for the low-rank structure in the correlated algorithm is similar to the MSE of the independent algorithm, indicating that adjusting the covariance structure helps the imputation performance.

We apply the rank-1 correlated model to the full data, with no validation set, to infer trends in microbiome diversity over time. We consider three approaches to generate uncertainty bounds for the mean diversity at each time point. For approach 1., we impute the missing data at their posterior mean, treat the values as fixed, and create 95% confidence intervals using the classical frequentist approach via a t-distribution. For approach 2., we use only the observed data at each timepoint, and create a 95% interval via a t-distribution. Note the t-interval for a mean is equivalent to a Bayesian credible interval with a uniform prior on the mean and log-uniform prior on the variance of the values. Thus, for approach 3. we use the posterior samples to propagate uncertainty from the imputation step and generate credible intervals for the full model. That is, let mean$\left(\alpha_{k,t}\right)$ and $sd\left(\alpha_{k,t}\right)$ be the sample mean and standard deviation for diversity at timepoint k and MCMC iteration t, including observed values and imputed values simulated from the posterior. Then, we simulate a value for the population mean ${\overset{‾}{\alpha }}_{k,t}$ via

$${\overset{‾}{\alpha }}_{k,t}=mean\left(\alpha_{k,t}\right)+sd\left(\alpha_{k,t}\right)T_{51}$$

where $T_{51}$ is a t-distributed random variable with 51 degrees of freedom. Intervals obtained via the quantiles of the ${\overset{‾}{\alpha }}_{k,t}$ will then properly account for both variability in the imputed values and sampling variability.

**Figure 1:**
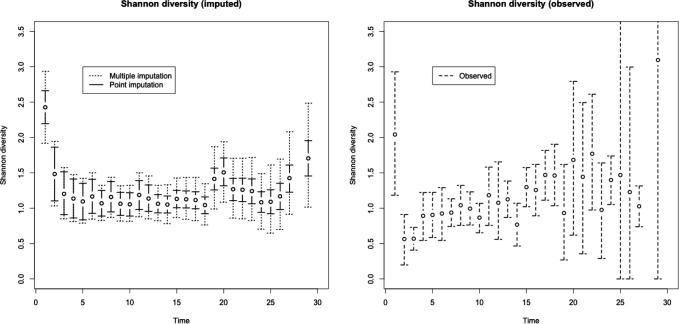
Estimates for Shannon diversity over time for the neonatal infant ClrX data. The left panel gives the mean under imputation and credible intervals generated using either point or multiple imputation, the right panel gives the mean and credible interval for observed data only.

The resulting trends for Shannon diversity, with 95% confidence/credible bounds, are shown in Figure 1. The confidence bounds generated using the point-imputed data are substantially more narrow than the bounds generated using multiple imputation in some instances, illustrating the danger of underestimating uncertainty if imputed data are treated as fixed and known. In contrast, the bounds generated using only the observed data are sporadic and very wide in some cased, illustrating the disadvantages of completely ignoring missing data. The multiple imputation approach is a principled compromise between these two extremes, and show a pattern in which diversity decreases over the first few days of life in the NICU and then stays relatively constant.

### 5.2 Mouse oral microbiome application

Here we consider a longitudinal study of the oral microbiome, where we examined saliva samples collected from mice induced with carcinogens. Throughout the study, we tracked 65 mice across 6 regular sampling intervals. Data were processed as in Section 5.1, yielding a tensor with dimensions of 65 × 168 × 6. Due to the unavailability of saliva samples for all mice at every time point, this tensor also has fiber-wise missingness with approximately 25% of the samples being absent.

We evaluate performance for fiber-wise missing imputation as in Section 5.1 through cross-validation, and for illustration purposes we also consider performance of entry-wise imputation in which we randomly hold an additional 25% of the observed entries in the tensor out.

The results are summarized in Table 5. For both scenarios, the Rank-1 Bayesian imputation approach with correlation structure performs better than or similarly to alternatives. The fiber-wise MSE is similar between the correlated and independent methods. However, the MSE for Shannon diversity and entry-wise imputation is improved by accounting for correlation, are improved by accounting for residual correlation. Moreover, the model with residual correlation structure has appropriate coverage rates for the Shannon diversity, whereas the model with independence has under-coverage. However, the Bayesian model with residual correlation structure has appropriate coverage rates for the two diversity measures, whereas the model with independence has under-coverage. This again illustrates the importance of accurately capturing the multivariate distribution to reflect uncertainty in downstream analyses. Note, however, that the correlated model performs substantially worse for higher ranks with fiber-wise missingness, and this is likely due to overfitting with the large number of parameters.

**Figure 2:**
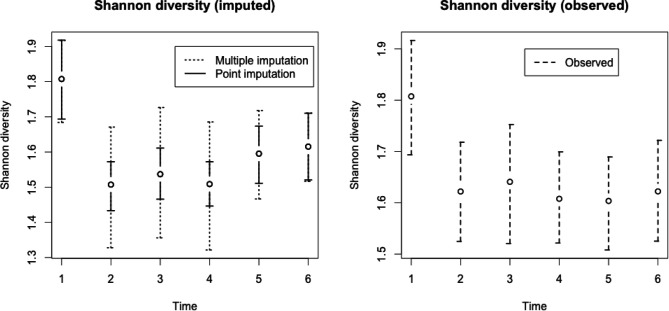
Estimates for mean Shannon diversity over time for the mouse application, with credible intervals generated using either point imputation, multiple imputation, or the observed data only.

We apply the rank-1 correlated model to the full data to infer trends in microbiome diversity, using the same approaches described in Section 5.1 of the manuscript. The resulting trends, with 95% confidence/credible bounds, are shown in Figure 2. The confidence bounds generated using the point-imputed data are again much more narrow than the bounds generated using multiple imputation, illustrating the danger of underestimating uncertainty if imputed data are treated as fixed and known. Nevertheless, the bounds generated using multiple imputation (and using only the observed data) still show significant changes over time. Namely, Shannon diversity decreases sharply after exposure, and there is some evidence for a rebound at subsequent timepoints but it is not conclusive.

## 6 Discussion

Our results demonstrate the advantages of accounting for residual covariance and uncertainty when imputing missing values in tensor data. While the motivating application for the development of BAMITA was longitudinal microbiome data, the model is generally applicable to a wide variety of application scenarios. Aspects of the model and sampling algorithm may be modified or extended, e.g., to capture spatiotemporal structure in relevant modes (Yokota et al., 2016; Guan, 2023) rather than a general covariance. Moreover, the assumption of normality for the residual error may be relaxed. For example, a tensor modeling approach is often effective for multi-condition RNA-Seq gene expression data (Hore et al., 2016), which may have a Poisson or negative binomial distribution.

In our data applications we selected the rank using cross-validation (Algorithm 2). Thus, subsequent analyses with the selected rank for the same data are prone to post-selection inference. However, as just one parameter (the rank) is empirically estimated, over fitting is not a major concern. For example, in our simulation, coverage rates are appropriate when the rank is selected through cross-validation. Nevertheless, a possible extension is to infer the number of components R (i.e., rank) as a parameter within the Bayesian model to account for its uncertainty, using reversible jump MCMC (Frühwirth-Schnatter et al., 2024) or other approaches. However, this would increase the computational complexity of the approach.

To make our algorithm applicable in various data application scenarios, we adopted the flat prior for the underlying parameters U which is independent of the data scale. However, under the specific data application scenarios, informative Gaussian priors can also be considered in our algorithm as a conjugate prior. One future topic is to extend our algorithm with the informative Gaussian priors on the underlying structure elements $U^{\left(1\right)},U^{\left(2\right)},\ldots ,U^{\left(N\right)}$.

In our real data applications, we use cross-validation to decide whether to adopt the Bayesian multiple imputation algorithm with the separable covariance structure or the independent structure, using mean squared error (MSE) of the imputed elements. However, alternative Bayesian model selection criteria, such as the deviance information criterion (DIC) (Spiegelhalter et al., 2002), could also be used to select the most appropriate model.

Computing time is often a bottleneck to fully Bayesian inference for high-dimensional data. We have carefully specified our models to facilitate efficient Gibbs sampling in high-dimensions. However, the model with independence error allows for a much more efficient algorithm; our largest dataset, described in Section 5.1, took several hours to run under the correlated model and under 10 minutes with independent error. Thus, this is a trade-off to modeling the residual covariance in higher dimensions.

## Figures and Tables

**Table 1:** Simulation results for study 1: rank selection and independent error. Results are presented as median values over all the missing elements. The best performance in each setting was marked in boldface.

Missing	Tensor	Missing	BAMITA independent				EM Algorithm	True	Selected	Overall
pattern	dimension	proportion	True Rank		Selected Rank		True Rank	Converged	Converged	Converged
		
			MSE	Coverage(%)	MSE	Coverage(%)	MSE	%	%	%

Entry Missing	(10× 10× 10)	20%	0.399	**94.8**	**0.327**	94.7	0.363	99	100	99
		50%	0.566	**95.2**	**0.508**	92.6	0.718	96	100	96
		70%	1.323	**93.0**	**0.869**	86.0	1.136	90	91	85
	(20× 20× 20)	20%	0.269	94.4	**0.267**	**94.5**	0.266	99	100	99
		50%	0.306	94.5	**0.302**	**94.7**	0.326	94	99	94
		70%	0.357	94.0	**0.335**	**95.0**	0.522	76	100	76
	(10× 100× 1000)	20%	**0.257**	94.4	**0.257**	**94.4**	0.259	97	97	97
		50%	**0.271**	94.4	**0.271**	**94.4**	0.272	98	98	98
		70%	**0.256**	94.3	**0.256**	**94.3**	0.287	92	92	91
Fiber Missing	(10× 10× 10)	20%	**0.375**	**95.3**	**0.375**	**95.3**	0.438	100	100	100
		50%	0.523	**93.1**	**0.478**	92.8	0.876	88	89	85
		70%	0.771	**87.3**	**0.641**	83.8	1.128	34	43	23
	(20× 20× 20)	20%	**0.285**	94.8	**0.285**	94.8	0.279	100	100	100
		50%	0.300	94.5	**0.298**	**94.8**	0.371	94	100	94
		70%	0.335	90.9	**0.335**	**93.1**	0.720	65	81	65
	(10× 100× 1000)	20%	**0.251**	**94.4**	**0.251**	**94.4**	0.265	100	100	100
		50%	**0.254**	**93.2**	**0.254**	**93.2**	0.346	98	98	97
		70%	0.260	**87.6**	**0.259**	**87.6**	0.570	52	48	47

**Table 2:** Simulation results for study 2: tensor imputation with correlated error. Results are presented as median values over all the missing elements.

Missing	Tensor	Missing	BAMITA Correlated		BAMITA Indep	missForest	EM	Corr	Inde	Overall
pattern	dimension	proportion	MSE	MSE low-rank	MSE	MSE	Algorithm	prop	prop	prop
			Coverage(%)	Coverage(%)	Coverage(%)		MSE	(%)	(%)	(%)

Entry Missing	(10× 10× 10)	20%	0.009	0.001	0.067	0.617	0.076	100	99	99
			97.4	98.2	94.4		0.076	100	99	99
		50%	0.033	0.007	0.064	0.914	0.110	100	92	92
			95.8	96.3	94.8		0.076	100	99	99
		70%	0.102	0.044	0.113	-	0.415	99	85	84
			93.6	93.7	94.6		0.076	100	99	99
	(20× 20× 20)	20%	0.007	0.001	0.105	0.371	0.105	100	100	100
			97.2	98.3	94.6		0.076	100	99	99
		50%	0.033	0.002	0.107	0.561	0.107	100	100	100
			95.9	97.3	94.8		0.076	100	99	99
		70%	**0.083**	0.009	0.117	0.886	0.151	100	82	82
			94.4	95.2	94.7		0.076	100	99	99
	(65× 168× 6)	20%	0.050	0.001	0.273	0.364	0.287	95	98	93
			95.8	97.3	94.3		0.076	100	99	99
		50%	0.138	0.005	0.275	0.397	0.277	90	93	84
			95.2	96.5	94.3		0.076	100	99	99
		70%	0.232	0.013	0.293	0.494	0.300	77	94	72
			94.6	95.9	94.3		0.076	100	99	99
Fiber Missing	(10× 10× 10)	20%	0.025	0.002	0.065	0.655	0.098	98	97	95
			94.5	98.4	94.7		0.076	100	99	99
		50%	0.085	0.017	0.083	0.988	0.339	75	64	54
			92.7	95.0	94.8		0.076	100	99	99
		70%	-	-	-	-	-	9	16	4
	(20× 20× 20)	20%	0.027	0.001	0.108	0.387	0.108	100	100	100
			93.2	98.3	94.8		0.076	100	99	99
		50%	0.073	0.003	0.112	0.595	0.145	96	88	85
			91.4	96.9	94.8		0.076	100	99	99
		70%	0.130	0.020	0.145	0.967	0.507	80	36	32
			90.2	92.4	94.4		0.076	100	99	99
	(65× 168× 6)	20%	0.109	0.002	0.286	0.421	0.283	82	99	81
			92.3	98.1	94.7		0.076	100	99	99
		50%	0.316	0.082	0.309	0.515	0.437	82	87	71
			91.9	96.0	94.4		0.076	100	99	99
		70%	0.798	0.538	0.555	0.904	0.761	59	49	33
			90.0	90.0	93.1		0.076	100	99	99

**Table 3:** Simulation results for study 3: imputation for a function of a fiber. Results are presented as median values over all the missing elements.

Missing	Tensor	Missing	BAMITA		BAMITA		EM Algorithm	Converged	Converged	Converged
pattern	dimension	proportion	Correlated		Independent		True Rank	Proportion	Proportion	Proportion
		
			MSE(Imputation)	MSE(Fiber)	MSE(Imputation)	MSE(Fiber)	MSE(Imputation)	Correlated	Independent	Overall
			Coverage(%)	Coverage(%)	Coverage(%)	Coverage(%)	-	-	-	-

Entry Missing	(10× 10× 10)	20%	0.409	-	**0.314**	-	0.322	100	100	100
			88.0	**78.0**	**95.0**	53.0	-			
		50%	0.396	-	**0.327**	-	0.423	100	97	97
			91.3	**90.0**	**95.0**	72.0	-			
		70%	0.596	-	**0.466**	-	0.776	98	99	97
			90.7	**89**	**94.9**	83.5	-			
	(20× 20× 20)	20%	0.266	-	**0.260**	-	0.265	100	100	100
			92.2	**91.2**	**94.8**	36.2	-			
		50%	0.284	-	**0.274**	-	0.282	100	100	100
			92.0	**91.2**	**94.9**	53.2	-			
		70%	0.278	-	**0.272**	-	0.326	100	86	86
			93.0	**91.0**	**94.9**	62.3	-			
	(65× 168× 6)	20%	0.287	-	**0.260**	-	0.270	100	99	99
			91.9	**91.8**	**94.8**	13.8	-			
		50%	**0.268**	-	0.271	-	0.299	100	98	98
			92.9	**92.6**	**94.8**	22.3	-			
		70%	**0.250**	-	0.278	-	0.305	100	96	96
			93.6	**92.1**	**94.7**	26.0	-			
Fiber Missing	(10× 10× 10)	20%	0.404	0.767	**0.311**	**0.617**	0.332	99	100	99
			90.0	**90.2**	**94.8**	83.0	-			
		50%	0.875	1.122	**0.514**	**0.915**	0.612	78	92	73
			89.5	**87.1**	**94.6**	82.8	-			
		70%	**2.609**	5.153	4.041	**3.510**	0.964	9	39	7
			86.6	83.8	**93.2**	**84.2**	-			
	(20× 20× 20)	20%	0.285	0.668	**0.266**	**0.616**	0.273	100	100	100
			93.0	**90.3**	**94.9**	67.9	-			
		50%	0.320	0.766	**0.285**	**0.701**	0.367	100	91	91
			92.3	**87.4**	**94.8**	68.4	-			
		70%	0.430	0.837	**0.369**	**0.769**	0.645	93	61	59
			91.8	**86.1**	**94.6**	69.6	-			
	(65× 168× 6)	20%	0.261	**0.940**	**0.259**	0.947	0.288	100	99	99
			94.6	**80.0**	**94.8**	29.4	-			
		50%	0.465	0.990	**0.337**	**0.958**	0.431	89	85	77
			92.9	**76.3**	**94.6**	31.0	-			
		70%	0.869	1.005	**0.540**	**0.979**	0.686	32	43	15
			90.7	**71.8**	**93.2**	32.0	-			

**Table 4:** Fiber-wise imputation results under cross-validation for the neonatal ClrX application. ’Imputation MSE’ gives relative MSE for held-out values in the tensor, ’low-rank MSE’ gives relative MSE when imputing via the low-rank term only in the correlated model, and ’Shannon MSE’ gives MSE for imputed Shannon entropy for held-out fibers. Coverage rates for 95% credible intervals are shown in parentheses.

Number of	BAMITA			BAMITA		EM Algorithm
components	Correlated			Independent		
	Imputation MSE	Low-rank MSE	Shannon MSE	Imputation MSE	Shannon MSE	Imputation MSE
	(Coverage)		(Coverage)	(Coverage)	(Coverage)	

1	0.340	0.583	0.744	0.560	1.834	0.569
	(95.4)		(84.3)	(94.1)	(55.3)	
2	0.345	0.570	0.698	0.533	1.471	0.557
	(95.3)		(84.4)	(94.3)	(55.0)	
3	0.353	0.561	0.665	0.518	1.035	0.554
	(95.1)		(84.2)	(94.5)	(63.0)	
4	0.365	0.547	0.635	0.505	0.924	0.543
	(95.0)		(81.9)	(94.5)	(63.3)	
5	0.384	0.552	0.611	0.505	0.903	0.555
	(94.8)		(82.0)	(94.3)	(61.7)	
6	0.391	0.547	0.608	0.497	0.874	0.554
	(94.6)		(81.8)	(94.2)	(60.0)	
7	0.423	0.576	0.608	0.496	0.827	0.555
	(94.3)		(80.9)	(94.1)	(59.7)	
8	0.439	0.578	0.605	0.499	0.794	0.554
	(94.2)		(80.8)	(94.1)	(59.1)	

**Table 5:** Imputation performance under cross-validation for the mouse ClrX application. ’Imputation MSE’ gives relative MSE for held-out values in the tensor, ’low-rank MSE’ gives relative MSE when imputing via the low-rank term only in the correlated model, and ’Shannon MSE’ gives MSE for imputed Shannon entropy for held-out fibers. Coverage rates for 95% credible intervals are shown in parentheses.

Missing	Number of	BAMITA			BAMITA		EM Algorithm
pattern	components	Correlated			Independent		
		Imputation MSE	Low-rank MSE	Shannon MSE	Imputation MSE	Shannon MSE	Imputation MSE
		(Coverage)		(Coverage)	(Coverage)	(Coverage)	

Random Missing	1	0.261	0.315	-	0.315	-	0.316
		(96.1)		-	(94.9)	-	
	2	0.259	0.304	-	0.295	-	0.306
		(96.0)		-	(94.9)	-	
	3	0.259	0.294	-	0.285	-	0.299
		(96.0)		-	(94.9)	-	
	4	0.268	0.297	-	0.280	-	0.296
		(96.0)		-	(94.9)	-	
Fiber Missing	1	0.338	0.341	0.117	0.339	0.169	0.338
		(96.6)		(97.5)	(94.8)	(87.0)	
	2	0.969	0.971	0.105	0.394	0.139	0.361
		(96.3)		(96.0)	(94.8)	(82.6)	
	3	3.291	3.293	0.151 1	0.632	0.129	0.377
		(94.4)		(90.8)	(94.9)	(81.6)	
	4	7.306	7.306	0.234	0.843	0.127	0.450
		(94.1)		(82.6)	(94.0)	(78.5)	

**Figure 3:**
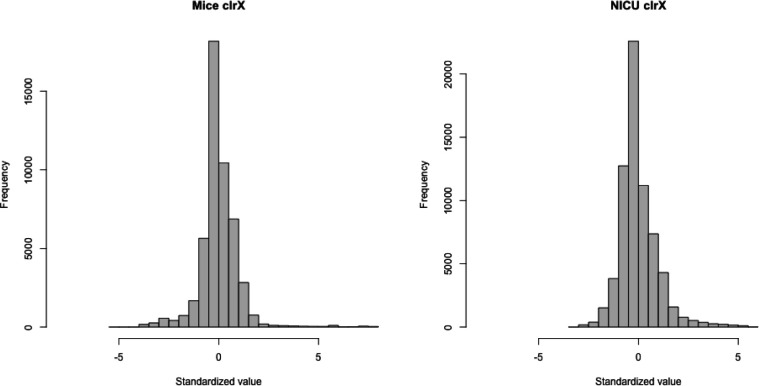
Histograms for the standardized data from the two case studies

**Figure 4:**
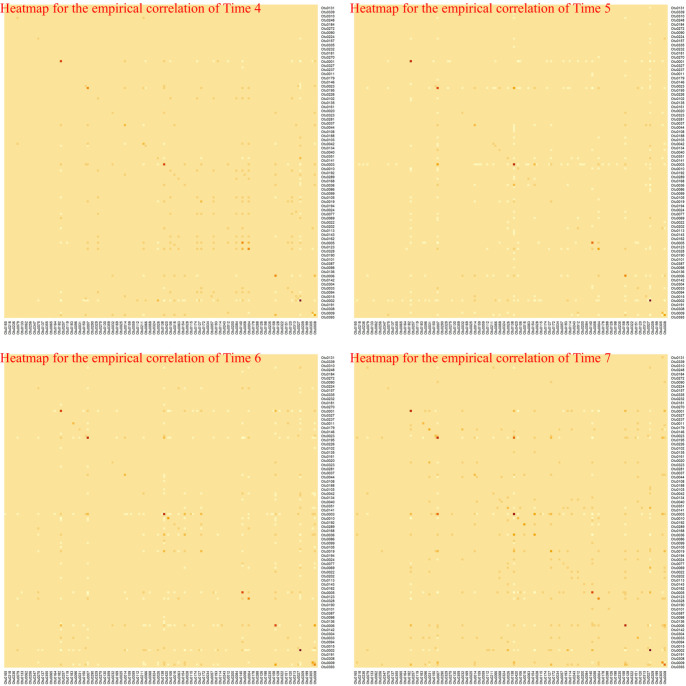
Heatmaps for the empirical taxa correlation matrix of the NICU example at different observation timepoints.

**Figure 5:**
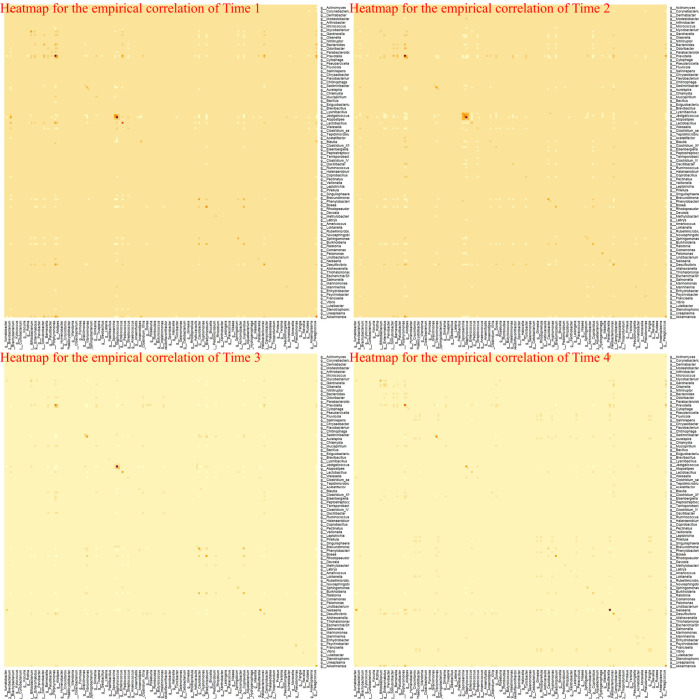
Heatmaps for the empirical taxa correlation matrix of the mice example at different observation timepoints.

## A Additional plots for microbiome applications

Here we present some plots for the data used in both microbiome applications presented. In particular, we check the appropriateness of the normality assumption and a separable covariance structure for both data applications.

We first present the histogram for the standardized observed data values for both the NICE data and the mice data. The histograms are present in Figure 3. From the figure, we can see that the histograms are generally centered around zero and exhibit a bell-shaped distribution without significant skewness. This suggests that, while the data may not be perfectly normal, the model with normally distributed errors can be a reasonable approximation for both datasets. This is further validated supported by the appropriate coverage rates for held-out data values shown for both applications.

In Figure 4 and 5, we present the heatmap for the correlation matrix of the observed data across taxa at different time points. From the figures, we can see that the heatmaps are very similar at different time points. The consistency of these heatmaps supports the assumption that the relative correlation structure remains the same over time, as is assumed for a separable covariance.
